# Effect of low-dose selenium on thyroid autoimmunity and thyroid function in UK pregnant women with mild-to-moderate iodine deficiency

**DOI:** 10.1007/s00394-014-0822-9

**Published:** 2014-12-19

**Authors:** Jinyuan Mao, Victor J. Pop, Sarah C. Bath, Huib L. Vader, Christopher W. G. Redman, Margaret P. Rayman

**Affiliations:** 1 Department of Nutritional Sciences, Faculty of Health and Medical Sciences, University of Surrey, Guildford, GU2 7XH UK; 2 Department of Endocrinology and Metabolism, The First Hospital of China Medical University, Shenyang, China; 3 Department of Clinical Health Psychology, Tilburg University, Tilburg, The Netherlands; 4 Clinical Laboratories, Máxima Medical Center Eindhoven, Veldhoven, The Netherlands; 5 Nuffield Department of Obstetrics and Gynaecology, John Radcliffe Hospital, University of Oxford, Oxford, UK

**Keywords:** Selenium, Iodine, Pregnancy, Thyroid autoimmunity, Thyroid peroxidase antibodies, Thyroid function

## Abstract

**Purpose:**

Selenium is an essential trace mineral and a component of selenoproteins that are involved in the production of thyroid hormones and in regulating the immune response. We aimed to explore the effect of low-dose selenium supplementation on thyroid peroxidase antibody (TPO-Ab) concentration and thyroid function in pregnant women from a mild-to-moderate iodine-deficient population.

**Methods:**

Samples and data were from a secondary analysis of Selenium in PRegnancy INTervention (SPRINT), a double-blind, randomized, placebo-controlled study that recruited 230 women with singleton pregnancies from a UK antenatal clinic at 12 weeks of gestation. Women were randomized to receive 60 µg/day selenium or placebo until delivery. Serum thyroid peroxidase antibodies (TPO-Ab), thyrotropin (TSH) and free thyroxine (FT4) were measured at 12, 20 and 35 weeks and thyroglobulin antibodies (Tg-Ab) at 12 weeks.

**Results:**

93.5 % of participants completed the study. Se supplementation had no more effect than placebo in decreasing TPO-Ab concentration or the prevalence of TPO-Ab positivity during the course of pregnancy. In women who were either TPO-Ab or Tg-Ab negative at baseline (Thy-Ab^−ve^), TSH increased and FT4 decreased significantly throughout gestation (*P* < 0.001), with no difference between treatment groups. In women who were Thy-Ab^+ve^ at baseline, TSH tended to decrease and was lower than placebo at 35 weeks (*P* = 0.050). FT4 fell more on Se than placebo supplementation and was significantly lower at 35 weeks (*P* = 0.029).

**Conclusions:**

Low-dose selenium supplementation in pregnant women with mild-to-moderate deficiency had no effect on TPO-Ab concentration, but tended to change thyroid function in Thy-Ab^+ve^ women.

## Introduction

Selenium (Se) is an essential trace element, which carries out its nutritional functions through the selenoproteins, 25 of which have been identified in humans [1]. Se is important for human health and affects immune and thyroid function [2]. The thyroid has the highest Se concentration of all tissues indicating its importance to that organ. It contains many selenoproteins including the deiodinases (Dio), the glutathione peroxidases, the thioredoxin reductases, selenoprotein P (SEPP1) and selenoprotein S; these are involved in regulating thyroid hormone metabolism and redox status [3].

The presence of thyroid autoantibodies to thyroid peroxidase (TPO-Ab) and thyroglobulin (Tg-Ab) is common in women of reproductive age and is indicative of thyroid inflammation and an increased risk of developing autoimmune thyroid disease, such as Hashimoto’s thyroiditis [4]. TPO-Ab positivity in euthyroid women is associated with a series of fetomaternal complications such as miscarriage, preterm delivery, postpartum thyroid dysfunction and even impaired neuropsychological development in the offspring [5–7]. In recent years, a number of studies have reported that Se supplementation decreased the concentration of TPO-Ab, but not all studies have shown benefit [8]. Considerable heterogeneity occurred among these studies with respect to age, gender, Se dose, baseline Se and iodine status, TPO-Ab concentration, thyroid function and whether the thyroid hormone, levothyroxine (LT4), was simultaneously administered. To date, only one study has been carried out in pregnant women; that study showed that Se supplementation during pregnancy and the postpartum period reduced TPO-Ab concentration, the incidence of postpartum thyroid dysfunction and permanent hypothyroidism [7]. Unfortunately, that study did not measure iodine status, which is crucial for thyroid function and may influence the effect of Se on the thyroid [9]. Several studies have reported that pregnant women in the UK are iodine deficient [10–12]. Normal thyroid function during pregnancy is important for a healthy pregnancy and fetal neurological development [13].

We had the opportunity to investigate the effect of a nutritional dose of Se on TPO-Ab concentration and thyroid function in pregnancy by using stored samples from the Se in PRegnancy INTervention (SPRINT) study in which iodine status was also measured [14, 15].

## Subjects and methods

### Participants

The SPRINT study [14] was a double-blind, randomized, placebo-controlled, single-center study (Registration no. ISRCTN37927591). It was conducted in accordance with the guidelines of the Declaration of Helsinki. All procedures involving human subjects were approved by the Milton Keynes Research Ethics Committee (REC reference no. 08/H0603/46). Written informed consent was obtained from all subjects.

Two hundred and thirty women in their first pregnancy were recruited at 12–14 weeks and randomized to receive 60 µg/day Se (as Se-yeast) or placebo-yeast until delivery. Blood samples were collected at 12 (baseline), 20 and 35 gestational weeks. Serum was prepared, and samples were stored at –80 °C until analyzed. One woman, recruited in error, was excluded from the analysis, leaving 114 women in the placebo group and 115 women in the Se group.

### Laboratory analyses

Whole-blood Se concentration at 12 and 35 weeks and urinary iodine concentration (UIC) at 12 weeks were measured by dynamic reaction cell inductively coupled plasma mass (SCIEX Perkin-Elmer, Beaconsfield, UK) [14, 16]. Urinary creatinine was measured by the Jaffe rate method, and individual iodine status was expressed as the iodine-to-creatinine ratio as previously reported [16]. Plasma SEPP1 concentration at 35 weeks was measured by ELISA [14, 17].

Serum thyroid-stimulating hormone (TSH), free thyroxine (FT4) and TPO-Ab at 12, 20 and 35 weeks were measured with commercial kits on Modular Analytics E170 (Roche Diagnostics, Germany); serum Tg-Ab at baseline was measured using the Cobas e601 analyzer (Roche Diagnostics, Germany). Inter- and intra-assay coefficients of variation for all measurements were less than 5 %. Thyroid dysfunction was assessed by trimester-specific reference ranges as established by Stricker et al. [18]. The criteria for various thyroid dysfunctions were defined as shown in Table 1.

**Table 1:** Criteria for diagnosis of various thyroid dysfunctions in each trimester

Category	Definition	Thyroid parameters	First trimester	Second trimester		Third trimester
Trimester-specific reference range [18]	2.5–97.5 percentile of reference population	TSH (mU/l)	0.07–2.82	0.33–2.89	0.32–2.94	
		FT4 (pmol/l)	10.48–18.49	9.4–14.06	8.5–13.54	
Overt hyperthyroidism	TSH below reference range and FT4 above	TSH (mU/l)	<0.07	<0.33	<0.32	
		FT4 (pmol/l)	>18.49	>14.06	>13.54	
Subclinical hyperthyroidism	TSH below reference range and normal FT4	TSH (mU/l)	<0.07	<0.33	<0.32	
		FT4 (pmol/l)	10.48–18.49	9.4–14.06	8.5–13.54	
Overt hypothyroidism	TSH above reference range and FT4 below	TSH (mU/l)	>2.82	>2.89	>2.94	
		FT4 (pmol/l)	<10.48	<9.4	<8.5	
Subclinical hypothyroidism	TSH above reference range and normal FT4	TSH (mU/l)	>2.82	>2.89	>2.94	
		FT4 (pmol/l)	10.48–18.49	9.4–14.06	8.5–13.54	
Isolated hypothyroxinemia	Normal TSH; FT4 below the lowest 10 percentile of reference population	TSH (mU/l)	0.07–2.82	0.33–2.89	0.32–2.94	
		FT4 (pmol/l)	<11.43	<9.97	<9.42	
TPO-Ab positivity (TPO-Ab^+ve^)	TPO-Ab above cutoff value	TPO-Ab (kU/l)	≥35	≥35	≥35	
Substantially elevated TPO-Ab positivity	TPO-Ab above cutoff value	TPO-Ab (kU/l)	≥100	≥100	≥100	
Tg-Ab positivity (Tg-Ab^+ve^)	Tg-Ab above cutoff value	Tg-Ab (kU/l)	>115	–	–	
Thyroid antibody positivity (Thy-Ab^+ve^)	Either TPO-Ab positivity or Tg-Ab positivity	Either TPO-Ab ≥ 35 kU/l or Tg-Ab > 115 kU/l		–		–

### Statistical analysis

Categorical variables between the two groups were analyzed with the Chi-squared test or Fisher’s exact test. The prevalence of TPO-Ab positivity within each group was analyzed by Cochran’s *Q* test. Continuous variables (whole-blood Se, plasma SEPP1, iodine-to-creatinine ratio and TPO-Ab concentration) with skewed distributions were analyzed by the Mann–Whitney *U* test between groups. Within groups, the Wilcoxon matched-pairs test was used.

As TSH was not normally distributed and included values between 0 and 1, a constant of 1 was added to the values that were then log-transformed (Log-TSH) to achieve normality; TSH values were reported as geometric means and 95 % confidence intervals (CIs) by back-transformation into the original units. Continuous variables (Log-TSH and FT4) with normal distribution were analyzed within subjects by a paired *t* test. A General Linear Model was used for comparison of Log-TSH and FT4 between groups, controlling for several covariates (continuous) at baseline, including age, gestational age at recruitment, BMI, log-transformed whole-blood Se (Log-Se) and log-transformed iodine-to-creatinine ratio (Log-Iodine). When exploring the effect of Se versus placebo on Log-TSH or FT4 at 20 and 35 weeks, baseline Log-TSH or FT4 was also added into the model as covariates, respectively.

Statistics were conducted using IBM SPSS statistics version 20. Tests of significance were two-tailed, and statistical significance was set at *P* < 0.05.

## Results

### Se status at baseline and after Se supplementation and iodine status at baseline

At baseline, whole-blood Se [median (IQR)] did not differ between Se and placebo groups [1.31 (1.19–1.46) vs. 1.32 (1.16–1.47) µmol/l, *P* = 0.565] either in the whole population [14], or in Thy-Ab^−ve^ women [1.31 (1.18–1.46) vs. 1.33 (1.15–1.48) µmol/l, *P* = 0.920] or Thy-Ab^+ve^ women, [1.32 (1.26–1.61) vs. 1.29 (1.15–1.44) µmol/l, *P* = 0.157]. At 35 weeks, whole-blood Se in the Se group was significantly higher than in the placebo group [1.87 (1.68–2.15) vs. 1.16 (1.05-1.30) µmol/l, *P* < 0.001], as was SEPP1 [5.30 (4.58–5.90) vs. 3.00 (2.30–3.60) mg/l, *P* < 0.001] [14].

At baseline, in the whole population, median (IQR) UIC was 42.0 (24.5-84.8) μg/l and median (IQR) iodine-to-creatinine ratio was 102.5 (67.3–166.8) μg/g. Iodine-to-creatinine ratio in the Se group was significantly lower than in the placebo group [92.5 (62.8–161.3) vs. 116.0 (75.5–182.0) μg/g, *P* = 0.049]. However, this significant difference only occurred in Thy-Ab^−ve^ women [94.0 (63.0–158.0) vs. 117.0 (73.25–190.25) μg/g, *P* = 0.034], not in Thy-Ab^+ve^ women [86.0 (62.3–179.8) vs. 97.5 (75.5–141.8) μg/g, *P* = 0.972].

### TPO-Ab concentration at baseline and after Se supplementation

At baseline, 25 (11.0 %) women were TPO-Ab^+ve^ with a median (IQR) TPO-Ab concentration of 110 (60–220) kU/l. In TPO-Ab^+ve^ women, TPO-Ab concentration at baseline was similar in the Se and placebo groups [120 (65–203) vs. 110 (57–230) kU/l, *P* = 0.89]. Though TPO-Ab concentration decreased over the course of gestation (*P* < 0.001), there was no difference in the magnitude of decrease between Se and placebo groups (54.2 vs. 65.6 %, *P* = 0.785), nor in the prevalence of TPO-Ab positivity at any gestational week (Table 2).

**Table 2:** Prevalence of thyroid dysfunctions in placebo and Se groups at different weeks of gestation

Gestational week	Placebo group		Se group		*P* value^a^
	No. in group	No. with dysfunction *n* (%)	No. in group	No. with dysfunction *n* (%)	
Tg-Ab > 115 kU/l					
12 weeks	110	11 (10)	113	11 (9.7)	0.947
TPO-Ab ≥ 35 kU/l					
12 weeks	113	15 (13.3)	115	10 (8.7)	0.269
20 weeks	112	12 (10.7)	109	8 (7.3)	0.382
35 weeks	109	7 (6.4)	105	6 (5.7)	0.828
TPO-Ab ≥ 100 kU/l					
12 weeks	113	8 (7.1)	115	6 (5.2)	0.757
20 weeks	112	7 (6.3)	109	3 (2.8)	0.333
35 weeks	109	3 (2.8)	105	0 (0)	0.247
Subclinical hypothyroidism					
12 weeks	113	12 (10.6)	115	10 (8.7)	0.623
20 weeks	112	13 (11.6)	108	7 (6.5)	0.186
35 weeks	109	13 (11.9)	106	16 (15.1)	0.479
Isolated hypothyroxinemia					
12 weeks	113	1 (0.9)	115	2 (1.7)	1.000
20 weeks	112	2 (1.8)	108	3 (2.8)	0.679
35 weeks	109	10 (9.2)	106	10 (9.4)	0.948

^a^ *P* values were from Chi-square test or Fisher’s exact test to compare placebo group *versus* Se group

### Prevalence of thyroid dysfunctions at baseline and after Se supplementation

One woman had overt hyperthyroidism and two had subclinical hyperthyroidism on recruitment, but none of these conditions was apparent by 20 and 35 weeks. There was no case of overt hypothyroidism. The prevalence of subclinical hypothyroidism (SCH) and hypothyroxinemia is shown in Table 2 and did not differ significantly between Se and placebo groups at any gestational week.

### Effect of Se supplementation on thyroid function

TSH at 12 weeks in Thy-Ab^+ve^ women was significantly higher than in Thy-Ab^−ve^ women [2.53 (2.12–2.98) vs. 1.30 (1.19–1.40) mU/l, *P* < 0.001], and FT4 was significantly lower [14.59 (14.20–14.97) vs. 15.15 (14.90–15.41) pmol/l, *P* = 0.016]. As thyroid antibodies influence thyroid function [19], the effect of Se supplementation on thyroid function was explored separately in Thy-Ab^−ve^ and Thy-Ab^+ve^ women (Table 3). At 12 weeks, TSH and FT4 did not differ between the Se and placebo groups in either Thy-Ab^−ve^ or Thy-Ab^+ve^ women.

**Table 3:** Thyroid function during gestation in the placebo and Se groups

Gestational week	Placebo group			Se group			*P* value^c^
	*n*	Unadjusted value^a^	Adjusted value^b^	*n*	Unadjusted value^a^	Adjusted value^b^	
*Thy-Ab* ^*−ve*^ *women*							
TSH (mU/l)							
12 weeks	95	1.30 (0.01, 4.20)	1.27 (1.12, 1.42)	98	1.30 (0.16, 4.00)	1.33 (1.19, 1.48)	0.567
20 weeks	93	1.63 (0.38, 4.03)	1.66 (1.57, 1.75)	94	1.67 (0.74, 3.67)	1.65 (1.56, 1.74)	0.897
35 weeks	91	1.85 (0.38, 4.98)	1.85 (1.74, 1.98)	90	1.96 (0.45, 5.35)	1.86 (1.74, 1.99)	0.917
*P* value^d^		<0.001			<0.001		
FT4 (pmol/l)							
12 weeks	95	15.29 (1.84)	15.30 (14.93, 15.67)	98	15.02 (1.76)	15.01 (14.65, 15.38)	0.256
20 weeks	93	12.68 (1.38)	12.61 (12.42, 12.81)	94	12.53 (1.51)	12.63 (12.43, 12.82)	0.879
35 weeks	91	11.19 (1.45)	11.13 (10.90, 11.36)	90	11.15 (1.34)	11.26 (11.03, 11.49)	0.303
*P* value^d^		<0.001			<0.001		
*Thy-Ab* ^*+ve*^ *women*							
TSH (mU/l)							
12 weeks	18	2.45 (0.91, 5.30)	2.46 (1.89, 3.13)	16	2.45 (1.00, 5.30)	2.61 (1.99, 3.36)	0.754
20 weeks	18	2.16 (1.08, 4.26)	2.27 (2.01, 2.55)	13	2.42 (1.43, 3.95)	2.43 (2.10, 2.78)	0.460
35 weeks	17	2.39 (1.34, 4.92)	2.50 (2.24, 2.79)	14	2.28 (1.02, 5.07)	2.10 (1.83, 2.38)	0.050
*P* value^d^		0.900			0.310		
FT4 (pmol/l)							
12 weeks	18	14.33 (1.08)	14.45 (13.87, 14,94)	16	14.88 (1.09)	14.80 (14.23,15.36)	0.334
20 weeks	18	12.38 (0.94)	12.53 (12.10, 12.96)	13	12.33 (0.96)	12.13 (11.62,12.63)	0.237
35 weeks	17	11.44 (1.23)	11.67 (11.03, 12.31)	14	10.82 (1.50)	10.54 (9.83, 11.25)	0.029
*P* value^d^		<0.001			<0.001		

^a^Unadjusted value of TSH was expressed as median (minimum, maximum) and FT4 as mean (standard deviation)

^b^Adjusted value of TSH was expressed as geometric mean (95 % CI) and FT4 as mean (95 % CI) with adjustment for the effect of covariates

^c^ *P* values were from General Linear Model comparing placebo and Se groups adjusted for covariates at baseline, including age, gestational age at recruitment, BMI, Log-Se, Log-Iodine and corresponding thyroid parameters, i.e., Log-TSH or FT4

^d^ *P* values were from paired t test comparing 12 to 35 weeks in each treatment group

In Thy-Ab^−ve^ women, TSH significantly increased during pregnancy in both Se and placebo groups (*P* < 0.001), with no difference between groups. By contrast, in Thy-Ab^+ve^ women on placebo, TSH decreased slightly from 12 to 20 weeks with a minor increase toward the end of gestation to give a level almost identical to that at 12 weeks (*P* = 0.900), though with a much narrower confidence interval (95 % CI range at 35 weeks, 2.24–2.79 vs. 1.89–3.13 at 12 weeks). In Thy-Ab^+ve^ women on Se, there was a gradual nonsignificant (*P* = 0.310) decrease in TSH that continued until term at which time it became almost significantly lower than in Thy-Ab^+ve^ women on placebo, after adjustment for baseline covariates (*P* = 0.050). Throughout gestation, TSH was significantly higher in Thy-Ab^+ve^ than in Thy-Ab^−ve^ women in both placebo and Se groups (all *P* < 0.01 at 12, 20 and 35 weeks).

In Thy-Ab^−ve^ women, FT4 decreased significantly from 12 to 35 weeks (*P* < 0.001), and the percentage drop was similar in both groups: 26.4 and 25.1 % in placebo and Se groups, respectively (*P* = 0.289). In Thy-Ab^+ve^ women, FT4 also fell significantly (*P* < 0.001), but the drop in magnitude in the placebo group was less than in the Se group (19.3 vs. 27.7 %, *P* = 0.012), resulting in FT4 at 35 weeks being significantly lower in the Se group (*P* = 0.029) after adjustment for baseline covariates.

We tested all two-way interactions between the treatment and confounders, including baseline iodine status (with outcome of Log-TSH and FT4 as the dependent variable); these were null (data not shown).

## Discussion

This is the first paper to explore the effect of a nutritional dose of Se on thyroid autoimmunity and thyroid function in pregnant women with mild-to-moderate iodine deficiency (based on WHO criteria [20]). We found that Se supplementation was no more beneficial than placebo in reducing TPO-Ab concentration, though it tended to influence thyroid function in Thy-Ab^+ve^ women.

The prevalence of TPO-Ab positivity in our population is similar to that reported in previous studies of women of childbearing age [6]. However, in contrast to the reduction in TPO-Ab concentration in Thy-Ab^+ve^ pregnant women on supplementation with Se found by Negro et al. [7], we found no such effect. There are a number of plausible reasons for the difference between two studies: (1) we only had 25, as opposed to 151, TPO-Ab^+ve^ women; thus, we had much less chance of seeing an effect; (2) the median baseline TPO-Ab concentration in our women was much lower: 110 versus 600 kU/l—a higher concentration appears to respond better to treatment [21]; (3) the dose we gave was much lower: 60 versus 200 μg/d; given the substantial fall (12 %) in blood Se in the placebo group in this trial from 12 to 35 weeks, which is likely due to the uptake of Se (as selenoprotein P) by placental receptors combined with an increase in plasma volume [14, 22], our Se dose was probably too low to sustain an effect; (4) none of our study participants received LT4 treatment in contrast to some 20 % of the TPO-Ab^+ve^ women in Negro’s study; as LT4 has an additive effect with Se in reducing TPO-Ab concentration in patients with Hashimoto’s thyroiditis (an autoimmune hypothyroid condition) [23], it may have helped reduce the TPO-Ab concentration in a percentage of those women.

The effects of Se supplementation on TSH and FT4 differ somewhat between women with and without thyroid antibodies. In our Thy-Ab^−ve^ women, the significant increase in TSH and decrease in FT4 from 12- to 35-week gestation reflect the physiological adaptations of the maternal thyroid during pregnancy and are in line with previous findings [24, 25]. The effect of fetal demands, physiological rise in T4-binding globulin and increased maternal plasma volume, may cause FT4 to drop progressively and by negative feedback, regulate the pituitary to produce more TSH to stimulate the thyroid [24]. The progressive increase in TSH during gestation may also reflect a stimulated thyroid state related to the relatively low iodine status of our population [24]. Se treatment had no effect on these changes.

By contrast, in the Thy-Ab^+ve^ group, where TSH was significantly higher, TSH did not increase in women on placebo and indeed tended to decrease in women on Se. The lack of increase in women on placebo reflects the immunosuppression associated with tolerance of the fetal allograft [26]. This immune-suppressed state can reduce autoimmunity and decrease the Thy-Ab concentration, as observed in our study, preventing an increase in TSH in Thy-Ab^+ve^ women [19, 27]. The finding that TSH decreased more in the Se group (*P* = 0.050) than in the placebo group suggests that Se supplementation may have an additive effect on the natural downsizing of the immune response, at least in TPO-Ab^+ve^ women. This can be understood by the potential of Se supplementation to reduce inflammation and modulate the immune response [7, 28]; Se may increase the number of regulatory T cells that can aid immune tolerance [29]. The fact that TSH did increase slightly in women on placebo toward the end of gestation (despite increasing immunosuppression) might also be explained by iodine deficiency or by the increasing uptake of Se by the placenta in late pregnancy [14, 22], resulting in an inadequate amount of Se remaining for the protection of the thyroid. However, at all assessments, TSH remained significantly higher in the TPO-Ab^+ve^ group compared to the TPO-Ab^−ve^ group.

FT4 declined significantly throughout gestation in all participants regardless of thyroid antibody status or treatment. However, FT4 dropped significantly more in Thy-Ab^+ve^ women on Se than in those on placebo. We suggest that this may simply be a reflection of the lower TSH in the Se-treated TPO-Ab^+ve^ group that resulted in lower stimulation of FT4 production in the thyroid.

Our study has a number of limitations. It was not designed to look at the effect of Se supplementation on thyroid function so these data result from secondary analyses. The main other limitation is the low sample size, which may have limited our power to detect significant differences. The strength of our study is that none of the women was being treated with LT4 (thyroid function is not routinely screened in pregnancy in the UK), which allowed us to observe the natural evolution of thyroid function and the effect of Se supplementation on TH in pregnant women with mild-to-moderate iodine deficiency.

As maternal T4 is essential for fetal development, further investigation is needed to confirm our findings and evaluate whether the higher FT4 drop in Thy-Ab^+ve^ women with mild-to-moderate iodine deficiency supplemented with Se is detrimental or whether it is compensated for by the decrease in TSH.
